# Clinical Characteristics and Microbiological Profile of Septic and Septic Shock Patients Admitted to the Intensive Care Department: A Single-Center Review

**DOI:** 10.7759/cureus.101658

**Published:** 2026-01-16

**Authors:** Mahmod Elshahat Makhlof, Hesham Kewan, Hussein Kandeel, Anand Kotgire, Mohammad Omar, Mohamed Shaheryar Ahmed, Nor Mahmod, Mohamed El Bouhy, Syed Ahmed Urooj

**Affiliations:** 1 Critical Care Medicine, Dubai Health - Hatta Hospital, Dubai, ARE; 2 Critical Care Medicine, Faculty of Medicine and Health Sciences, University of East Anglia, Norwich, GBR; 3 Critical Care Medicine, Faculty of Medicine, Gulf Medical University, Ajman, ARE

**Keywords:** blood culture, epidemiology, microbiology, sepsis, septic shock

## Abstract

Objectives

The study objectives include determining the incidence of different microbial isolates and the prevalence of multidrug-resistant microbes retrieved from the blood of septic and septic shock patients.

Design and setting

A retrospective observational study was conducted to explore the clinical and bloodstream microbiological characteristics of all sepsis and septic shock patients who required critical care services in Hatta Hospital - Dubai Health between January 2018 and December 2023. The primary focus was to characterize the microbiological profile of septic patients and identify their characteristics in relation to a short-term (28 days) outcome.

Patients above the age of 13 admitted to Hatta Hospital intensive care services with clinical suspected sepsis or septic shock were enrolled. A total of 246 patients were enrolled. The study population age ranged from 13 to 112 years, with a median of 81 years; there were 108 females (43.9%) and 138 males (56.1%).

Results

The blood culture was positive in 53 (21.5%) of patients, but negative-culture sepsis was reported in 193 (78.5%) patients. The most common isolated species were gram-negative bacteria, including *Escherichia coli*, *Klebsiella pneumonia,* and *Pseudomonas aeruginosa*. With regard to the early 28-day mortality, the survivors were 178 (72.4%) and the nonsurvivors were 68 (27.6%). Fifty-eight (85.3%) of the recorded early mortality was noted in blood culture-negative patients, while ten (14.7%) recorded early mortality was noted in blood culture-positive patients.

Conclusions

Blood culture-negative sepsis and septic shock demonstrated a higher early mortality compared to blood culture-positive patients.

## Introduction

The latest definition of sepsis is a “life-threatening organ dysfunction due to a dysregulated host response to infection” with a subsequent self-organ injury [1]. It is generally accepted that the earlier one can identify the diagnostic markers for sepsis, they are able to introduce interventions earlier and produce better clinical outcomes. However, the greatest challenge has been to identify the most effective clinical biomarker. Generally, lactate is considered the most effective biomarker in sepsis diagnosis; however, there are two major flaws to using this as a standalone diagnostic tool. One is that most academics agree that lactate is diagnostic of sepsis when the value is greater than 2; however, by the time this is achieved, the patients clearly septic already and thus management can be initiated regardless of the biochemical abnormality, and the second flaw is that lactate can be raised due to a variety of other factors such as current medication use or tissue hypoperfusion that is not related to sepsis [2,3]. Thus, there has been a race to identify the use of other inflammatory cytokines that may be beneficial adjuncts to lactate. C-reactive protein (CRP) has been identified as a biomarker of some use; however, it is generally accepted that there is a delayed phase of CRP reaction in sepsis, and thus, by the time the CRP is raised, the patient is clearly septic [4]. Procalcitonin has become the intermediate step for most nations, pioneered primarily in the United States; however, cost and uncertain prognostic measures have restricted its global adoption [5,6].

With the uncertainty in the diagnostic viability of biomarkers to predict sepsis, there is a scope for accepting this limitation and identifying other avenues to assist in effective sepsis management. This was the goal of the Surviving Sepsis Campaign: accepting that three- and six-hour bundles were ineffective in good clinical care and thus introducing the "hour-1 bundle" for the immediate commencement of resuscitation and management [7]. There has also been a directive to produce clinical assessment tools to help guide early sepsis management. By assessing the host's response to infection and organ dysfunction, academics have aimed to develop scoring tools such as the National Early Warning Score (NEWS-2) or Sequential Organ Failure Assessment (SOFA) scores to promote early sepsis management and early escalation of care as appropriate [8]. These scoring tools have allowed for the initiation of early broad-spectrum antibiotics, which have resulted in a 33% reduction in all-cause sepsis mortality [9]. However, there is a growing fear among the academics that while this method is effective in reducing mortality in the short term, the lowered threshold for initiation of broad-spectrum antibiotics is accelerating the development of antibiotic resistance [10]. Best seen during the COVID-19 pandemic, antibiotic initiation thresholds were lowered even further, a short-sighted management strategy. There is a not-so-distant future where the broad-spectrum antibiotics available to us become ineffective [11].

With this in mind, there is a rapid need for hospitals to identify the pathogen responsible for infection and switch to specific targeted antibiotic therapy. Early identification of a site of infection is imperative to effective management; however, patients and pathogens are globally ambiguous, so identifying one source of infection is often challenging. Thus, epidemiological knowledge is invaluable to aiding microbiologists and clinicians in switching to appropriate antibiotics and management regimens early on. There is an accepted limitation in the delay in identifying sepsis early through biomarkers, so there is an anecdotal acceptance of this limitation with a shifted focus on early and effective microbiology treatment. The goal of intensive care units (ICUs) should be to produce rubrics and frameworks specific to their institutions that combine clinical characteristics of patients with the known epidemiology of their region to produce local algorithms for best care. The goal of this paper is to present the best strategy we have found for producing such frameworks in our ICU and present the epidemiological data of our region for other ICUs to gain insight from.

## Materials and methods

Aims and objectives

This study aims to identify the clinical and bloodstream microbiological characteristics of septic and septic shock patients requiring critical care services in Hatta Hospital, Dubai Health, Dubai, United Arab Emirates. The frequencies of microbiological isolates retrieved from the blood of this patient cohort and the corresponding antimicrobial sensitivities were correlated to the clinical phenotypes, with the aim of developing an algorithm to develop and evaluate future protocols and interventions that could be implemented into the ICU setting to improve sepsis management and create awareness of local epidemiological trends.

The study objectives include determining the incidence of different microbial isolates and the prevalence of multidrug-resistant microbes retrieved from the blood of septic and septic shock patients, as well as defining the local pattern of resistance and antibiogram sensitivities for the most common microbial blood growth in critically ill patients in our region.

Definitions

Sepsis was identified by suspected or confirmed infection AND an organ dysfunction as defined by a SOFA [12]. Septic shock was identified by either a vasopressor requirement to maintain a mean arterial pressure (MAP) of 65 mm Hg or greater or a serum lactate level greater than 2 mmol/L (>18 mg/dL) [13]. Multidrug resistance (MDR) was defined as the presence of (i) methicillin or quinolone or glycopeptide resistance for any type of *Staphylococcus*; (ii) glycopeptide resistance for any type of Enterococcus; (iii) macrolide or glycopeptide resistance for *Streptococcus pneumoniae*; (iv) extended-spectrum β-lactamase (ESBL) inhibitor-associated semi-synthetic or synthetic penicillin or third-generation cephalosporin or quinolone or carbapenem resistance for any Gram-negative organism; or (iv) triazole or polyene resistance for any type of isolated *Candida *spp. or yeast [14]. Early mortality was defined by death within four weeks of sepsis-related ICU admission [15].

Primary outcome

The primary focus was to characterize the microbiological profile of septic patients and identify the characteristics of this population in relation to a short-term (28 days) outcome.

Secondary outcome

The secondary outcome was to assess the ICU-related deaths and hospital-related morbidities. Morbidities were defined as the consequences or complications other than death that resulted from their condition.

Population

This retrospective observational study was conducted on all patients admitted to Hatta Hospital's intensive care department with a diagnosis of sepsis or septic shock between January 2018 and December 2023.

The study was approved by the Dubai Scientific Research Ethics Committee (DSREC-08/2024-05), Dubai Health.

Inclusion Criteria

All patients above the age of 13 admitted to Hatta Hospital's intensive care services with clinically suspected sepsis or septic shock from 01/01/2018 to 31/12/2023 were included.

Exclusion Criteria

Patients admitted to intensive care who did not have clinically suspected sepsis, were under the age of 13, or who did not consent to their data being used for this study were excluded.

Data collection

Data was sourced from the medical records of patients on the Epic Electronic Health Record (EHR) system of the United Arab Emirates database of medical records. An MS Excel spreadsheet (Microsoft Corp., USA) was created that contained patient demographic data, associated comorbidities, etiology of sepsis (respiratory, abdominal, urinary, soft tissue, or catheter-related), Acute Physiology and Chronic Health Evaluation (APACHE II) score, 24-hour SOFA score, mode of admission (emergency department (ER), wards, operative theater (OT)), laboratory values, blood cultures microbiology results, antimicrobial sensitivities, and multidrug-resistant isolates. A second spreadsheet was produced to characterize patient hospital management, including empirical versus targeted antimicrobial therapy, other associated therapies, mono versus combination antimicrobial therapy, frequencies of usage of individual antimicrobials, duration of antimicrobial therapy, culture-focused escalation, mechanical ventilation/vasopressors use, 28-day mortality, and ICU length of stay.

Statistical analysis

Data analysis was done using IBM SPSS Statistics for Windows, Version 29.0 (released 2022, IBM Corp., Armonk, NY). Quantitative variables are presented as mean and standard deviation (SD) or median and interquartile range (IQR) for non-parametric data. Qualitative variables are presented as counts and percentages. Paired-samples t-test was used to compare quantitative variables at two different time points, a Student's t-test (or Mann-Whitney U test) was used to compare quantitative data between two independent groups, and one-way analysis of variance (ANOVA) test (or Kruskal-Wallis test) was used for the comparison of more than two groups. The Chi-square test (or Fisher's exact) was used to compare qualitative data between different groups. Pearson’s and Spearman’s correlation tests were used to measure linear correlation between different quantitative variables. All statistical variables were determined, with P < 0.05 being statistically significant.

## Results

Patient demographic data and comorbidities

A total of 317 records were assessed, after which 246 patients were eligible for inclusion. The age range was from 13 to 112 years, with a median age of 79. Gender split was 108 females (43.9%) and 138 males (56.1%). Hypertension and diabetes mellitus were the most common comorbidities reported in the studied patients. The respiratory system was the most common source of sepsis, with pneumonia emerging as the most common diagnosis. The breakdown of patient demographics and site of infection can be seen in Table 1, and the breakdown of patient scoring and biochemical markers can be seen in Table 2.

**Table 1:** Patients’ demographic data and comorbidities ICU: intensive care unit, ER: emergency room, CKD: chronic kidney disease, IHD: ischemic heart disease, COPD: chronic obstructive pulmonary disease

Variable	Median (range)/(%)
Age	79 (99)
Sex	
Male	138 (56.1%)
Female	108 (43.9%)
Venue	
Ward	149 (60.5%)
ER	89 (36.1%)
OT	8 (3.2%)
Site of infection	
Respiratory	128 (52.0%)
Urinary tract	49 (19.9%)
Skin/soft tissue	19 (7.72%)
Abdominal	17 (6.91%)
Catheter- related	3 (1.22%)
More than one site	30 (12.2%)
Community-acquired	183 (74.4%)
Hospital-acquired	45 (18.3%)
ICU-acquired	18 (7.3%)
Comorbidities	
Diabetes mellitus	139 (56.5%)
Hypertension	173 (70.3%)
IHD	109 (44.3%)
Heart failure	61 (24.8%)
COPD	56 (22.8%)
CKD	70 (28.5%)

**Table 2:** Patients’ overall evaluation APACHE: Acute Physiology and Chronic Health Evaluation, CRP: C-reactive protein, ICU: intensive care unit, LOS: length of stay, PCT: procalcitonin, PLT: platelet count, WBC: white blood cell count

Variable	Median	Range
APACHE II score	18	41
PCT (ng/ml)	12.72	38.34
CRP (mg/L)	132.7	111.93
WBC (103/uL)	11.962	7.5
PLT (103/uL)	229.87	111.76
Highest s. Lactate in ICU day 1	1.95	18.8
ICU-LOS /sepsis episode duration	9	82

Microbiology findings

Of the 246 patients, 53 (21.5%) had positive blood cultures, while 193 (78.5%) patients had negative. The most common isolated species were gram-negative bacteria, including *Escherichia coli*,* Klebsiella pneumonia,* and *Pseudomonas aeruginosa*. Five patients had polymicrobial growths in their cultures. The breakdown of culture findings can be seen in Table 3.

**Table 3:** Patients’ microbiological profile CRE: Carbapenem-resistant Enterobacteriaceae, ESBL: extended-spectrum β-lactamase, MRSA: methicillin-resistant *Staphylococcus aureus*

Blood culture-negative	193 (78.5%)
Blood culture-positive	53 (21.5%)
Bacterial sepsis	40 (75.47%)
Fungal sepsis	13 (24.53%)
MRSA	7 (2.8%)
ESBL	29 (11.8%)
CRE	5 (2%)
AmpC	6 (2.4%)
Resistant candida	8 (3.3%)
Blood culture growth species	
Escherichia coli	12 (22.64%)
Pseudomonas aeruginosa	6 (11.32%)
Klebsiella pneumonia	6 (11.32%)
Klebsiella aerogenes	2 (2.377%)
Candida albicans	4 (7.54%)
Candida auris	4 (7.54%)
Candida glabrata	4 (7.54%)
Candida parapsilosis	1 (1.88%)
Staphylococcus capitis	4 (7.54%)
Staphylococcus aureus	3 (5.66%)
Staphylococcus epidermidis	1 (1.88%)
Streptococcus pneumonia	1 (1.88%)
Providencia stuartii	1 (1.88%)
Stenotrophomonas melophilia	1 (1.88%)
Enterococcus faecalis	3 (5.66%)
Enterococcus faecium	1 (1.88%)
Campylobacter coli	1 (1.88%)
Burkholderia cepacian	1 (1.88%)
Bacteroides fragilis	2 (3.77%)
Polymicrobial growth	5 (9.43%)

Patient outcomes

In regard to the early 28-day mortality, 178/246 (72.4%) were survivors, and 68/246 (27.6%) passed due to their sepsis. Of these 68 patients, 10/68 (14.7%) were from the positive blood culture group. The breakdown can be seen in Table 4. Antimicrobial therapy was initiated empirically in 206/246 patients (84.1%) and was initiated as targeted sensitivity therapy in 40/246 (16.3%) patients. Meanwhile, 226/246 (91.9%) patients were immediately started on broad-spectrum antibiotics, with 154 (68.1%) started on IV piperacillin-tazobactam and 72 (31.9%) started on meropenem. Out of the 154 uses of piperacillin-tazobactam, 110/154 (71.4%) were used as the first-choice antibiotic coverage. Out of the 72 meropenem uses, 30/74 (40.5%) were initiated as the first-choice antibiotic. Ceftriaxone was used as the first-choice antibiotic on 62 (25.20%) occasions. The breakdown of antimicrobial treatment can be seen in Table 5. 

**Table 4:** Patients’ mortality and morbidity outcomes P-value < 0.05 is statistically significant.

	Median	Range	
ICU length of stay (LOS)	9	82	
	No. (% of the total population of 246)		
Morbidity	168 (68.3%)		
28-day mortality	68 (27.6%)		
	Survivor (n = 178)	Non-survivor (n = 68)	p-value
Blood culture positive	43 (24.1%)	10 (14.7%)	0.107
Blood culture negative	135 (75.9%)	58 (85.3%)	0.121

**Table 5:** Antimicrobial/other ICU treatments CRRT: continuous renal replacement therapy

	No.	%
Antibiotic/antifungal		
Piperacillin tazobactam	154	62.60%
Meropenem	72	29.30%
Ceftriaxone	62	25.20%
Levofloxacin	27	10.97%
Azithromycin	26	10.56%
Linezolid	23	9.34%
Cefepime	9	3.65%
Tigecycline	9	3.65%
Ciprofloxacin	8	3.25%
Ertapenem	6	2.43%
Vancomycin	5	2.03%
Ceftazidime	5	2.03%
Ampicillin	4	1.62%
Amoxycillin clavulanate	3	1.21%
Colistin	3	1.21%
Cefuroxime	2	0.81%
Ceftazidime avibactam	2	0.81%
Gentamicin	2	0.81%
Amikacin	2	0.81%
Ceftolozane tazobactam	1	0.40%
Caspofungin	23	9.35%
Voriconazole	4	1.62%
Fluconazole	3	1.21%
Anidulafungin	1	0.40%
More than one type	135	54.80%
Vasopressors needed	96	39%
CRRT needed	8	3.30%
Mechanical ventilation	160	65%
Empirical antibiotic	207	84.10%
Targeted antibiotic	40	16.30%
Mono-antimicrobial therapy	157	63.80%
Combination antimicrobial therapy	84	34.10%

Comparison between blood culture-positive and culture-negative patients

The studied patients were divided into the blood culture-positive group (N = 53) and the blood culture-negative group (N = 193). There were no significant differences between the biochemical markers in the blood culture-positive and negative groups. The breakdown of the findings between the blood culture-positive and -negative groups can be found in Table 6.

**Table 6:** Comparison between blood culture-positive and -negative groups. a: Pearson Chi-square, ***Chi-square test (FE: Fisher's exact). P-value < 0.05 is statistically significant.

	Blood culture-positive sepsis		Blood culture-negative sepsis		Mann-Whitney U	p-value
	(n =53)		(n=193)			
	Mean (SD)		Mean (SD)			
Age	73.62 ± 19.41		76.02 ± 23.08		4341	0.092
APACHE II	19.09 ± 6.87		17.56 ± 8.14		4584	0.247
24 hr. SOFA	5.96 ± 3.09		5.90 ± 3.90		4777.5	0.46
Highest s. Lactate in ICU-Day 1	3.11 ± 2.93		3.02 ± 3.03		5088.5	0.955
WBC (103/uL)	12.65 ± 10.65		11.77 ± 6.40		4898	0.638
PLT count (103/uL)	210.62 ± 113.08		235.16 ± 111.11		4441	0.142
CRP (mg/L)	143.93 ± 99.08		129.62 ± 115.26		4360	0.1
PCT (ng/ml)	11.71 ± 18.95		13.00 ± 42.17		3891.5	0.008
ICU length of stay	14.47 ± 14.12		13.03 ± 13.21		4905	0.647
	N	%	N	%	X2***	p-value
Mechanical Ventilation	34	64.20%	126	65.30%	0.024a	0.878
CRRT need	2	3.80%	6	3.10%	0.683	0.544
28-day mortality	10	18.90%	58	30.10%	2.600a	0.107
Vasopressors needed	26	49.10%	70	36.30%	2.857a	0.091

Comparison between survivors and non-survivors

The comparison between the survivor and non-survivor groups revealed a statistically significant difference regarding age, APACHE II score, SOFA score, highest serum lactate on ICU day 1, mechanical ventilation, and vasopressor requirement. The median survivor APACHE II score was 16, while the median non-survivor score was 86 (p = 0.007). The median SOFA score of the survivor group was 4, while the non-survivor median score was 8 (p < 0.001). Moreover, 95/178 (53.4%) required mechanical ventilation in the survivor group, while 57/68 (95.6%) required ventilation in the non-survivor group (p < 0.001). The breakdown of the comparison between the survivors and non-survivors can be seen in Table 7.

**Table 7:** Comparison between survivors and non-survivors a: Pearson Chi-square, APACHE: Acute Physiology and Chronic Health Evaluation, SOFA: sequential organ failure assessment. ‡ Mann-Whitney-U test presented as median and interquartile range. P-value < 0.05 is statistically significant.

	Survivors	Non-survivors	Mann-Whitney U	p-value
	(n= 178 - 72.4%)	(n= 68 - 27.6%)		
	Median (range)	Median (range)		
Age	79 (98)	86 (98)	4708	0.007
APACHE II score	16 (41)	23 (35)	3519	<0.001
24 hr. SOFA score	4 (15)	8 (13)	2838.5	<0.001
Highest s. Lactate in ICU day 1	1.800 (11)	3.550 (18.8)	3171	<0.001
ICU - LOS	8.00 (71)	12.00 (82)	4940	0.026
PCT (ng/ml)	0.6650 (253)	1.0900 (267.85)	4829	0.014
CRP (mg/L)	94.750 (548)	111 (391.9)	5602.5	0.368
WBC (10^3^/uL)	11 (63.9)	10.950 (34.7)	5758.5	0.557
PLT (10^3^/uL)	215.500 (709.4)	180.000(523)	5644	0.414
	N (%)	N (%)		
Blood culture positivity	10 (18.9%)	43 (81.1%)	2.600^a^	0.107
Mechanical ventilation	95 (53.4%)	65 (95.6%)	38.568^a^	<0.001
CRRT needed	4 (2.2%)	4 (5.9%)	0.222	0.15
Vasopressor needed	39 (21.9%)	57 (83.8%)	79.263^a^	<0.001

Comparison between sepsis and septic shock patients

Finally, the comparison between sepsis and septic shock groups revealed a statistically significant difference found regarding the APACHE II score, SOFA score, highest serum lactate in ICU day 1, blood procalcitonin level, mechanical ventilation and vasopressor requirement, and 28-day mortality. The median APACHE II score of the sepsis group was 16, while the median APACHE II score of the septic shock group was 23 (p < 0.001). The median SOFA score was 4 in the sepsis group and 8 in the septic shock group (p < 0.001). Moreover, 22/169 (13.0%) died within 28 days in the sepsis group, while 46/77 (59.7%) died in the septic shock group (p < 0.001). The breakdown of findings between the septic and septic shock patients can be seen in Table 8.

**Table 8:** Comparison between the sepsis and septic shock patients a: Pearson Chi-square, FE: Fisher exact, CRRT: continuous renal replacement therapy, LOS: length of stay. P-value < 0.05 is statistically significant.

	Sepsis	Septic shock	Mann-Whitney U	p-value
	(n = 169; 68.7%)	(n = 77; 31.3%)		
	Median (range)	Median (range)		
Age	79 (99)	79 (98)	6260.5	0.634
APACHE II score	16 (31)	23 (39)	3431.5	<0.001
SOFA score within 24 h	4 (15)	8 (15)	2489.5	<0.001
WBC (10^3^/uL)	10 (40.7)	11.800(65.6)	5828.5	0.19
Highest s. Lactate in ICU day 1	1.700 (10.4)	2.800 (18.8)	3542	<0.001
PLT (10^3^/uL)	217.000 (590.4)	196.000 (709.4)	6300.5	0.691
CRP (mg/L)	87.900 (548)	130.000 (379.3)	5456.5	0.042
PCT (ng/ml)	0.5800 (253.00)	1.3500 (267.87)	4682.5	<0.001
ICU - LOS	8.00 (71)	10.00 (82)	6012	0.338
	N (%)	N (%)		
Mechanical ventilation	88 (52.1%)	72 (93.5%)	39.943^a^	<0.001
CRRT needed	2 (1.2%)	6 (7.8%)	7.343^a^	0.07
Vasopressors needed	22 (13.0%)	74 (96.1%)	153.464^a^	<0.001
28-days mortality	22 (13.0%)	46 (59.74%)	130.992	<0.001

## Discussion

Sepsis represents a global health problem and a significant cause of critical illness-related morbidity and mortality. In the European Sepsis Occurrence in Acutely Ill Patients II (SOAP II) trial, septic shock was the most frequent cause of shock, with a hospital mortality in excess of 40% [16]. Sepsis and septic shock are medical emergencies that require treatment and resuscitation immediately, and the literature clearly points toward early identification and appropriate management in the initial hours, leading to better clinical outcomes.

In our study, the blood culture-positive and -negative groups were compared. We observed that the blood culture-negative patients were older with higher blood procalcitonin levels and higher levels of other biomarkers, indicative of poor prognosis. Although these were not statistically significant differences, it is an interesting difference to note, and perhaps in an extended population, these differences may become significant. While we cannot say for certain in our population that the negative blood culture group will absolutely have worse clinical outcomes, there is a potential discussion point here.

Through the work we have done in this paper, we have aimed to understand the microbiological phenotypes of sepsis and septic shock patients in our ICU, as well as begin to quantify the baseline characteristics of this population that could serve as an indicator of patient prognosis and outcome in our ICU and other similar units in our region in the future. We note several significant differences between survivors and non-survivor groups, particularly where the non-survivors exhibited statistically significant differences regarding age, APACHE II score, SOFA score, highest serum lactate on ICU day 1, mechanical ventilation, and vasopressor requirement. Looking at these trends, we hope to be able to improve our practices within our unit to better identify patients who may require more pertinent interventions earlier on, and by assessing such characteristics, we can identify patient populations in whom more targeted research may need to be done.

We also note that our findings are not absolute and that not every biomarker can be relied on to determine patient outcomes. When looking at the relationships between blood culture-positive and -negative groups, we found no statistically significant relation regarding the APACHE II score, the highest ICU day 1 arterial lactate levels, the SOFA score recorded within 24 hours after ICU admission, invasive ventilation/CRRT use, or ICU LOS. Although there was no significant difference in these biochemical or scoring markers, we did note significant differences in the 28-day mortality rate. Fifty-eight of the 193 patients in the non-blood culture-positive group died within 28 days, while only 10/53 died in the blood culture-positive group. This was a statistically significant difference, which suggests that identifying epidemiological strains early on is beneficial to patient outcomes and reduces overall mortality. This discrepancy highlights the impact of blood bacterial load on patient outcomes noted in our data, showing that the culture-negative severe sepsis group demonstrated significantly more comorbidities, acute organ dysfunction, and in-hospital mortality (34.6% vs. 22.7%; P < 0.001), while CRRT was less frequent (5.3% vs. 6.1%; P < 0.001).

The findings from our paper are in keeping with other literature in this field. Chang et al. reported a higher mortality in culture-negative septic shock patients (37.6% vs. 45.1%, p = 0.029) in their population and also noted that in-hospital mortality between culture-negative and culture-positive sepsis was reduced, although not significantly (31.6% vs. 34.9%, p = 0.073) [17]. Phua et al. reported a 41.5% of culture-negative severe sepsis patients and a 58.5% of culture-positive patients in a prospective observational cohort study of 1,001 ICU patients. The culture-negative patients had a lower procalcitonin levels, lower APACHE II (median 25.0 (interquartile range 19.0 to 32.0) vs. 27.0 (21.0 to 33.0), P = 0.001), SOFA scores, less need for vasoactive agents, and a shorter duration of hospital stay (12 days (7.0 to 21.0) vs. 15.0 (7.0 to 27.0), P = 0.02) [18].

We appreciate that there are limitations to the work we have produced. We note that our study includes a single center and that, too, a more rural area of the country. While this is a natural limitation to applicability to broader audiences, it does also address a major gap in the literature regarding the underrepresentation of rural centers and their epidemiology [19]. We also note that our paper has a limited population of a few hundred. While this may seem small in relation to the greater populations, it is notable that for the area of coverage of our hospital, it represents a respectable population. Through works such as these, it is possible to determine the epidemiology of more rural areas and help in producing region-specific protocols.

Overall, we hope that this article will help to present the demographic and microbiological characteristics of sepsis and septic shock patients admitted to our intensive care unit and how the findings from our population could help to predict patient outcomes and morbidity during ICU stay. We have shown that blood culture-negative sepsis and septic shock patients demonstrated a higher early mortality compared to blood culture-positive patients. We have discussed the microbiological strains noted in this population and the treatment regimens started, including their relative effectiveness in producing favorable patient outcomes. We hope this work will fill a gap in the epidemiological literature of our region and may help to justify and produce improved local guidelines in sepsis and septic shock management.

## Conclusions

In our study, the blood culture-negative sepsis and septic shock demonstrated a higher early mortality compared to blood culture-positive patients. We found that the blood culture-negative patients were older with a higher level of inflammatory biomarkers, more comorbidities, and in-hospital mortality. The blood culture positivity or negativity in sepsis and septic shock patients was not significantly related to the APACHE II score, the highest ICU day 1 arterial lactate levels, the SOFA score recorded within 24 hours after ICU admission, invasive ventilation/CRRT use, or ICU LOS. We think that the demographic and microbiological characteristics of sepsis and septic shock patients admitted to intensive care could help to predict and produce favorable patient outcomes during ICU stay. Further research is recommended to fill a gap in the epidemiological literature in sepsis and septic shock management.
